# Outcome comparison of Lisfranc injuries treated through dorsal plate fixation versus screw fixation

**DOI:** 10.1590/1413-78522014220600576

**Published:** 2014

**Authors:** Sun-jun Hu, Shi-min Chang, Xiao-hua Li, Guang-rong Yu

**Affiliations:** 1. Tongji University, School of Medicine, Yangpu Hospital, People's Republic of China, Department of Orthopedic Surgery, Yangpu Hospital, Tongji University School of Medicine, People's Republic of China; Tongji University, School of Medicine, Tongji Hospital, People's Republic of China, Department of Orthopedic Surgery, Tongji Hospital, Tongji University School of Medicine, People's Republic of China

**Keywords:** Tarsal joints, Arthrodesis, Internal fixators, Bone screw

## Abstract

**OBJECTIVE:**

The objective of this prospective study was to test whether the treatment of Lisfranc injuries with open reduction and dorsal plate fixation would have the same or better functional outcomes as treatment with standard trans-articular screw fixation.

**METHODS:**

Sixty patients with primarily isolated Lisfranc joint injury were treated by open reduction and dorsal plate fixation or standard screw fixation. The patients were followed on average for 31 months. Evaluation was performed with patients' chief complaint, clinical examination, radiography, and AOFAS Midfoot Scale.

**RESULTS:**

Thirty two patients were treated with open reduction and dorsal plate fixation, and twenty eight patients were treated with open reduction and screw fixation. After two years follow-up, the mean AOFAS Midfoot score was 83.1 points in the dorsal plate fixation group and 78.5 points in the screw fixation group (p<0.01). Of the dorsal plate fixation group, radiographic analysis revealed anatomic reduction in twenty-nine patients (90.6%, 29/32) and nonanatomic reduction in three patients. Of the screw fixation group, radiographic analysis revealed anatomic reduction in twenty-three patients and nonanatomic reduction in five patients (82.1%, 23/28).

**CONCLUSIONS:**

Open reduction and dorsal plate fixation for a dislocated Lisfranc injury do have better short and median term outcome and a lower reoperation rate than standard screw ORIF. In our experience, we recommend using dorsal plate in ORIF on dislocated Lisfranc injuries.** Level of Evidence II, Prospective Comparative Study.**

## INTRODUCTION

Ligament injuries and fracture-dislocations (Lisfranc injury) involving the tarsometatarsal (TMT) joints may lead to chronic pain and functional loss because of arthritis, deformity, residual ligamentous instability, and associated soft-tissue injury. The causes of the injury include low energy sports injuries and high energy crush injuries.^1^^,^ 2 Because of varying degree of violence, the injury may be purely ligamentous or associated with fractures of the metatarsals, cuneiforms, navicular and cuboid.^1^^-^ 3 Around 20% of Lisfranc fracture-dislocations are misdiagnosed or missed during the initial evaluation.^4^ This makes early and accurate diagnosis a prerequisite for appropriate management of these injuries in order to avoid long-term sequelae and functional impairment. The general consensus of Lisfranc injuries is that anatomical reduction and rigid stabilization of the Lisfranc joint through operation is imperative for good outcome.^3^^,^ 5 Conservative management with closed reduction and plaster immobilization does not appear to have an ideal outcome in the contemporary treatment of Lisfranc injuries because the initial reduction is always hard to achieve for soft tissue folder may block in the articular interspace, which only applied for a small portion of patients.^1^^,^ 3 ^,^ 4

A variety of treatments that have been advocated for Lisfranc injuries exist currently. There are advocates for both closed and open K-wire fixation after either closed or open anatomic reduction. The pins are left in for 6-8 weeks. More authors advocate the use of screw fixation to stabilize the disrupted joints, leaving them across the joints for 3 to 6 months. Open reduction using one or two parallel incisions in the dorsum of the foot and small cortical screws application seems to be the preferred method of management for the injuries in the joints of the medial and middle column (first, second and third metatarsals), while K-wires can be used for the stabilization of the lateral column (fourth and fifth metatarsals) in case of instability. From the standpoint of stability of fixation, screws appear to be superior at holding a reduction over multiple K-wires in mechanical tests.^6^ A recent interest has developed in suture-button fixation which is relatively fast, minimally invasive, and it eliminates the need for subsequent implant removal when treating the primary Lisfranc injuries.^7^ However, Ahmed's cadaveric study shows standard transarticular screw fixation with a 4.0mm cannulated screw had less displacement than the suture-button in isolated Lisfranc ligament injuries.^7^ He believes open reduction and screw fixation should continue to be the accepted treatment. Using miniplates to fix one or more column of the Lisfranc injuries has being advocated in recent years, so as to achieve rigid stabilization. Wilson and Gomez-Tristan,^8^ Aronow^9^ and Cosculluela *et al*.^10 ^have reported using miniplates in treating Lisfranc injuries and the short and medium term outcome was relatively good, but long-term outcome still needs to be confirmed. Dorsal plate fixation has the merit of avoiding iatrogenic injury of articular cartilage, but it requires a larger exposure which may increase the chance of infection, so it still requires long term evaluations.

The traumatic arthritis postoperation has been recognized as a complication that seriously influences the life quality of patients. It seems that anatomic reduction has not insured good functional outcomes. Recent series about treatment of Lisfranc injuries have reported AOFAS midfoot scores ranging from 65 to 77, with radiographic changes of traumatic arthritis present in up to 94% of cases.^11^ All of these series used transarticular screw fixation to maintain the well-reduced joint positions. Typically, 3.5mm or 4.0mm screws were used, and two or more screws were placed across the TMT joints. These finding has inspired a recent series advocating primary arthrodesis as a strategy for primarily Lisfranc injuries.^12^^,^ 13 Two prospective randomized studies have been reported, and Ly and Coetzee^13^ report concluded a primary stable arthrodesis of the medial two or three rays appears to have a better short and medium-term outcome than open reduction and screw fixation of Lisfranc joint injuries. Whether a primary stable arthrodesis is necessary, especially to those ligamentous injuries, is still controversial. Remove the whole cartilage surface and fix the joint can lead to the loss of the motion, since the TMT1 has minimal motion ability. Is it worthwhile? An ideal treatment method for Lisfranc injuries should reliably achieve a near-normal anatomic relationship between the midfoot articulations, holding these relationships until healing of ligament and bone occurs, while minimizing iatrogenic cartilage damage, and dorsal plate maybe an alternative in treating Lisfranc injuries.

The report of treatment for Lisfranc injuries with dorsal plates is relatively rare, especially for the report of clinical outcome comparing open reduction with dorsal plates fixation and screws fixation. The purpose of this prospective study was to test the treatment of Lisfranc injuries with primary open reduction and internal dorsal plate fixation would have the same or better functional short and medium-term outcomes as treatment with standard trans-articular screw fixation.

## MATERIALS AND METHODS

We performed this prospective study comparing two groups of patients with Lisfranc injuries. The study design was to include all injuries within three weeks from the date of injury and most had it within the first week from the date of injury. Patients were identified at the time of hospital admission and the information was collected. Inclusion criteria of patients: adult single Lisfranc injuries including ligamentous and/or osseous injuries or both. In order to reduce statistical bias, all the operations were done by one group of surgeons. Patients with these criteria were excluded: Pathologic fracture; polytraumatic patients associated with any other substantial foot, ankle, or leg injury; severe internal diseases which cannot undergo operation, such as insulin-dependent diabetes *mellitus*, coronary heart disease with congestive heart failure; ipsilateral ankle fusion; peripheral vascular disease; peripheral neuropathy; and rheumatoid arthritis.

**Figure 1:**
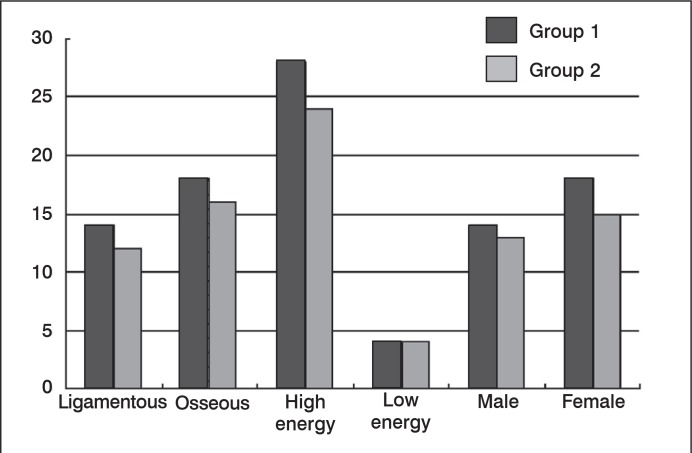
Details about the types of injuries and the causes of injuries.

Between March 2006 and June 2010, 62 patients were admitted to our institute with Lisfranc injuries and 60 patients underwent surgical intervention. (Figure 1) The mean age of the patients was 46 (20-72) years old. Group 1 is plate fixation group, while Group 2 is screw fixation group. In the dorsal plate fixation group, one or more column of TMT joints were open reduced and fixed by one or more mini plates according to severity of the injuries, while in the other group, medial and middle column were fixed through conventional way with formal open reduction and transarticular screws internal fixation. Standard 3.5 or 4.0 mm cortical screws were utilized depending on the patient's size. The fourth and fifth rays were reduced and stabilized with temporary Kirschner-wire fixation.

Patients were revisited at six weeks, three months, six months, one year and two years after operation, with clinical and radiographic assessment of the progress of healing and complications. We evaluated the outcomes with patient's chief complaint, clinical examination, and radiography. The functions of the injured feet were evaluated by the American orthopedic foot and ankle society clinical rating Scale (AOFAS Midfoot Scale).

### Surgical Technique

All the patients underwent routine laboratory tests and organ function assessment after admission. To the people who were extremely weak or had heart or pulmonary diseases, routinely arterial blood gas analysis was done as the baseline assessment. Internal diseases were actively treated. The time of the operation was strictly controlled until the swelling dissipated and the sign of skin fold can be seen. The mean operation time of the patients was 11 days after fracture (range 8 - 14 days).

Surgery was performed with the patient lying supine on a fracture table under general anesthesia or epidural anesthesia. A non-sterile tourniquet is applied to the proximal thigh, and the leg is prepped in sterile manner to the knee. A fluoroscopy unit is available for assessment of the injury, reduction, and hardware placement.

**Figure 2:**
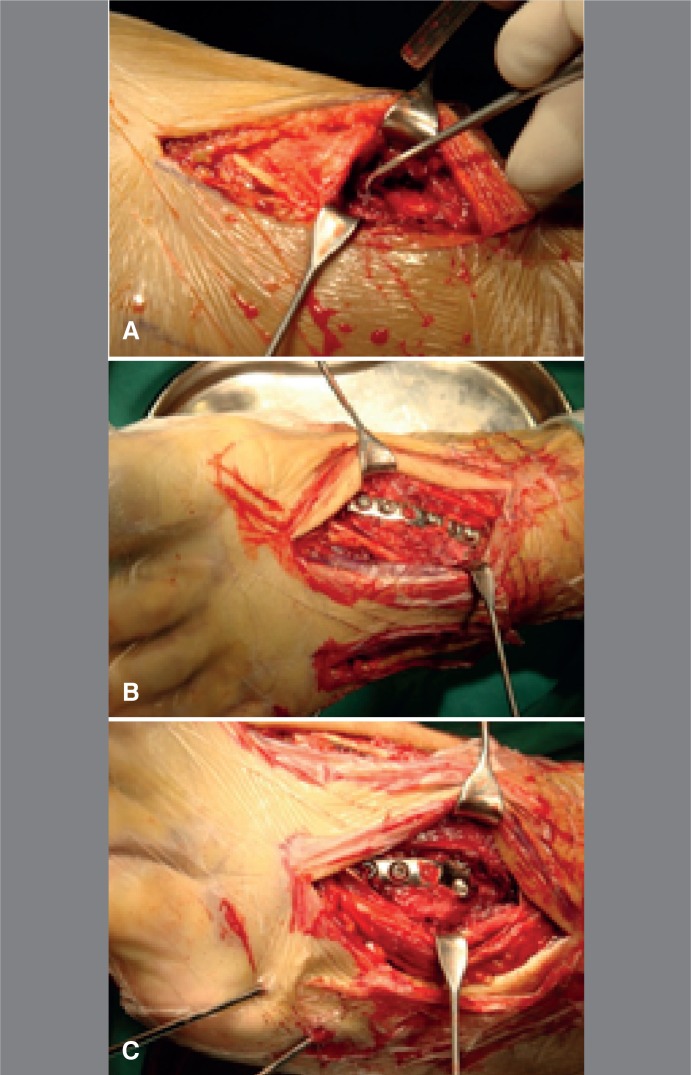
(A) The periosteum of first TMT joint is incised longitudinally to expose the dislocation, as well as the second TMT joint and the intercuneiform articulation. (B) The first TMT joint is fixed with a dorsal miniplate. (C) The third TMT joint is also fixed by a suitable miniplate.

After sterilization and cover with aseptic towel the tourniquet is inflated. An initial closed reduction should be done for complete dislocations to reduce the tensile pressure on the overlying skin and protect the soft tissues from further compromise. A longitudinal incision is made over the dorsomedial aspect between the first and second TMT joint. This incision may be approximately 5cm long which is allowed for visualization of both first and second TMT joint and the Lisfranc area. Care is taken to identify and protect the medial branch of the superficial peroneal nerve. Surgical approach takes the interval between the extensor hallucis longus and the anterior *tibialis* tendons with the two tendons drag bilateral sides and protected. The *dorsalis pedis *artery and deep peroneal nerve should also be protected. The periosteum of first TMT joint is incised longitudinally to expose the dislocation, as well as the second TMT joint and the intercuneiform articulation. (Figure 2) Once visualization of all involved medial joints is obtained, a thorough debridement of the joints is performed to remove any interposed tissue. Reduction and fixation is carried out with the assistance of fluoroscopy. Temporary Kirschner wire fixation is used before plate fixation when the fracture dislocation is difficult to maintain. However, in most cases we do not use it. The plates we use are mini plates including various shapes, such as "L" and "T" plates. The distal portion of plate is drilled and fixed with screws on the first metatarsal, and with the holding of position the proximal portion of plate is drilled and fixed to medial cuneiform. Care was taken to avoid placing the mini plates in the course of extensor tendons especially under the extensor to the great toe. The second and third TMT joint is also fixed by a suitable plate. (Figure 2) The second incision is made according to reduction needs, which may center between the fourth and fifth metatarsals, and the fourth or fifth TMT joint can be fixed through this incision by plate. (Figure 2) The plates are removed six to eight months postoperative.

One or two dorsal longitudinal incisions were made-one between the first and second metatarsals and the second centered between the fourth and fifth metatarsals. Open reduction and screw fixation of the first, second, and third metatarsal-cuneiform joints was performed. Then, whenever necessary, Kirschner wires were placed in each of lateral two rays. The Kirschner wires were removed between four and six weeks postoperatively. The screws were not routinely removed unless they caused symptoms, and they were removed six to eight months postoperative.

### Postoperative Management

Postoperatively, all patients wore a short leg splint for two weeks followed by a short leg cast for four to six weeks, and all remained non-weight-bearing during this time. If the patient is accompanied with metatarsal or cuneiform fractures, the time maybe delayed to eight weeks. The patients then are slowly advanced to full weight bearing in a rigid pneumatic boot device over the next four weeks. Physical therapy was started at six to ten weeks and included gait-training, swelling control, and range-of-motion exercises. Intense exercise and strengthening activities were allowed until all of the hardware is removed at the 6-month to 8-month postoperative interval.

## RESULTS

All the patients were followed-up for 2-3 years (average 31 months). No patients died or lost during this period. The severity of the patients conditions are shown in Table 1 and the Myerson classification is used. There are four patients with open fractures, three patients underwent open reduction and screw fixation and one patient underwent open reduction and dorsal plate fixation after the wound healed. There is no one who had bilateral injuries.

**Table 1:** Concrete distribution of the patients according to Myerson classification (No.).

	I	IIA	IIB	IIIA	IIIB
Group 1	8	6	12	4	2
Group 2	7	4	11	4	2

There was no patient with deep infection or osteomyelitis. Three patients with superficial infection were observed (9.4%, 3/32) and cured by oral antibiotic therapy and changing dressings in group 1, while there were two cases of superficial infection were observed (7.1%, 2/28) in group 2 without further surgery interference. Four patients had fringe of incision partially necrosis which partly prolonged the time of incision healing (12.5%, 4/32) in group 1, while there were three patients who had fringe of incision partially necrosis (10.7%, 3/28) in group 2. Two people underwent secondary arthrodesis in group 1 (6.3%, 2/32), while three people underwent secondary arthrodesis in group 2 (10.7%, 3/28). Overall, twenty-six patients (81.3%) were satisfied with the result and returned to their pre-injury works and activities in group 1, while twenty-two patients(78.6%) were satisfied with the result and returned to their pre-injury works and activities in group 2.

**Figure 3:**
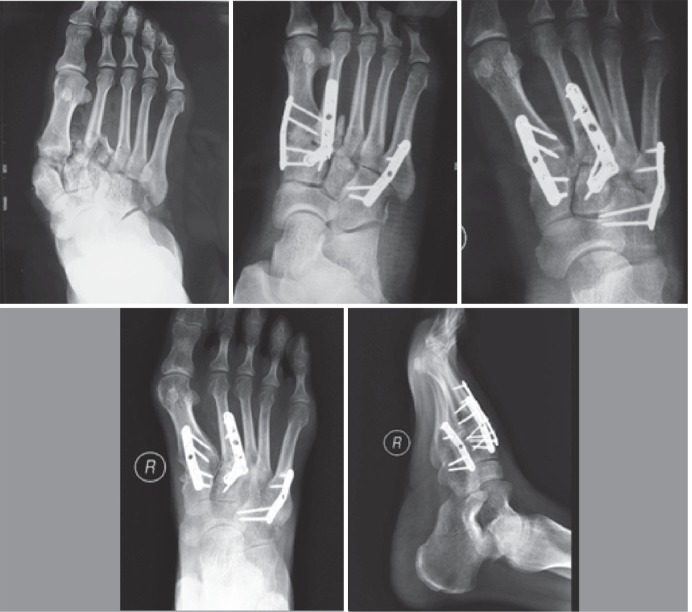
Case 1: Female 38 yer as old Myerson I type, X-radiograph before operation; X-radiograph shows the fracture-dislocation healed two weeks and six months post-operation.

**Figure 4:**
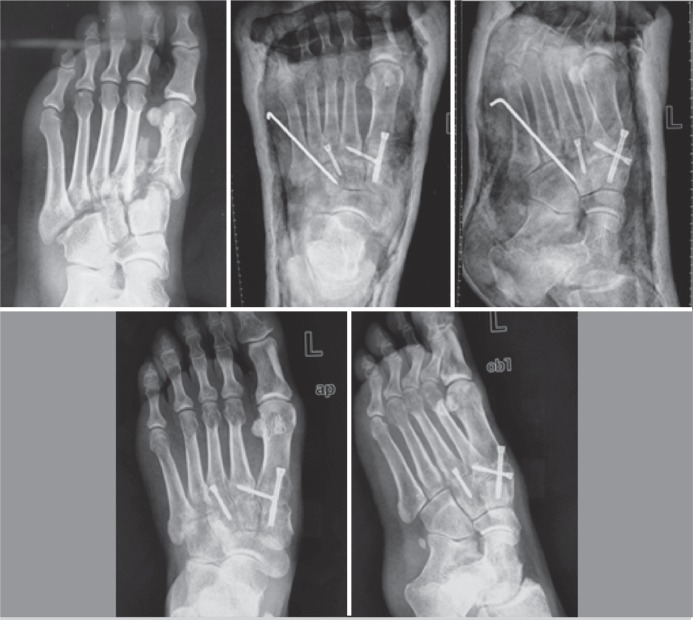
Case 2: Male 40 years old, Myerson IIB type, X-radiograph before operation; X-radiograph shows the fracture-dislocation healed two weeks and six months post-operation.

Functional outcome was measured by the AOFAS Midfoot Scale (Table 2). In group 1, the final mean score was 83.1(range, 41-100). Thirteen patients had excellent outcome (score≥90); Fourteen patients had good outcome (90>score≥75); Four patients had fair outcome(75>score≥50) and one patients had poor outcome (score<49). Six patients had mild discomfort on prolonged walking and five patients had reduced mobility subjectively. None of the patients were using modified footwear at final follow-up. A typical case with Myerson I type fracture-dislocation treated with dorsal plates is seen Figure 3. In group 2, the final mean score was 78.5 (range, 38-100). Eight patients had excellent outcome; thirteen patients had good outcome; four patients had fair outcome and three patients had poor outcome. The three people who had poor outcome also underwent secondly arthrodesis of TMT joints. Ten patients had mild discomfort on prolonged walking and nine patients had reduced mobility subjectively. A typical case with Myerson IIB type fracture-dislocation treated with screws and Kirschner wire is seen Figure 4. After 2 years follow-up the final AOFAS score in dorsal fixation group is higher than screw fixation group (p<0.01) .

**Table 2:** Comparison of dorsal group and screw group by AOFAS scores over time.

	3 months	6 months	12 months	24 months
Group 1	43.8	65.3	71.4	83.1
Group 2	37.5	59.1	64.2	78.5
p value	<0.05	<0.05	<0.01	<0.01

Radiographic analysis revealed anatomic reduction in twenty-nine patients and non-anatomic reduction in three patients in group 1(90.6%, 29/32). Five patients had spontaneous TMT joints fusion verifying from first to all fine rays, and four of the patients did not had any discomfort. There were eight patients with degenerative joint disease in different levels after removal of the inner plant (25.0%, 8/32). In group 2, radiographic analysis revealed anatomic reduction in twenty-three patients and non-anatomic reduction in five patients(82.1%, 23/28); six patients had spontaneous TMT joints fusion, and two patients had severe discomfort and pain. There were thirteen patients with degenerative joint disease in different levels after remove of the inner plant (46.4%, 13/28). One patient had one of transarticular screws broken but the injury healed anatomically at last and he had mild pain after prolonged walking. Two patients had undergone a tarsometatarsal arthrodesis while in group 2 there were three patients.

## DISCUSSION

Lisfranc (tarsometatarsal) joint injuries comprise only 0.2% of all injuries, most of which are closed injuries. These injuries can be osseous, ligamentous, or a combination of both. Quenu and Kuss first proposed the classification used by many today. It essentially divides these injuries into three types based on the resultant pattern. Hardcastle *et al*.^14^ further categorized Lisfranc injuries into Types A, B, or C based on displacement and incongruity with a system that they thought would dictate treatment. Myerson *et al*.^4^ followed with modifications to this system based on direction of dislocation. The recent AO/OTA classification also classifies these injuries by the resulting deformity. Despite these multiple classification schemes, outcome and treatment do not reliably correlate with any injury type.

The controversies on treatments of closed Lisfranc injuries include the method of reduction, the method of fixation and the need for primary arthrodesis in severe injuries.^12^^-^ 14 A variety of treatments which have been advocated for these injuries exists currently.^15^ Open reduction using one or two parallel incisions in the dorsum of the foot and screws fixation seems to be the preferred method of management for the injuries in the joints of the medial and median column (first, second and third TMTs), while K-wires can be used for the stabilization of the lateral column (fourth and fifth TMTs) in case of instability. Arntz *et al*.^16 ^reported on 34 patients with TMT fracture dislocations treated with open reduction and screws fixation. At an average of 3.4 years follow up, 27 of 29 patients (93%) who initially presented with closed injuries, reported an excellent or good outcome. However, there are several disadvantages of screw fixation for Lisfranc injuries. Since the screws are transarticular, their placement further damages the articular cartilage of the joints that an attempt is being made to preserve. Screw breakage can occur, and the distal portion of the screw is difficult to remove and is potentially left behind. Because of the risk of screw breakage, early foot and ankle range of motion exercises and weight bearing may be delayed, which may potentially delay a patient functional recovery time.^17^ The most serious problem is that the incidence of posttraumatic arthritis is relatively high, which may serious influence the patients' quality of life quality.^11^

Using plates to fix one or more column of the Lisfranc injuries has being advocated in recent years, so as to achieve rigid stabilization. Plates can provide rigid fixation without further damaging the articular surface. In a cadaver model for neuropathic TMT joint fusion, Marks *et al*.^18^ compared the plates and screws for TMT joints fusion. The plated constructs were shown to be stiffer and had less displacement at initial loading. None of the plated constructs failed during cyclic loading, while three out of eight screw constructs did. After cyclic loading, the plated specimens had significantly less displacement after fatigue loading and higher load to failure. Another cadaver study by Alberta *et al*.^17^ compared one fourth tubular plates applied dorsally with transarticular stainless screws across the first and second tarsometatarsal joints. After sectioning the Lisfranc and TMT joint ligaments, measurements were repeated in the loaded condition. No significant difference was noticed with direct comparison between plates and screws with respect to the ability to realign the first and second TMT joints and to maintain TMT joint alignment during loading. Transarticular screws and dorsal plates showed similar ability to reduce the first and second TMT joints after TMT and Lisfranc ligament transection and to resist TMT joint displacement with weightbearing load. Clinically, Wilson and Gomez-Tristan,^8^ Aronow^9^ and Cosculluela *et al*.^10^ have reported using mini plates in treating Lisfranc injuries and the short and medium term outcome was relatively good. However, the report on long-term outcome of treating Lisfranc injuries with dorsal plates is rare, and there is no prospective report about comparing dorsal plate fixation and standard screw fixation.

A larger exposure is required for insertion of the mini plate used in this technique when compared to insertion of percutaneous screws across the tarsometatarsal and intercuneiform joints, which may increase the chance of incision infection.^10^ In our study, three patients with superficial infection were observed (9.4%, 3/32) in group 1, while there were two cases of superficial infection observed (7.1%, 2/28) in group 2. However, open reduction has been recommended even in those cases of Lisfranc injury where anatomic reduction can be achieved by closed means or with the aid of percutaneous clamps.^7^ Open reduction allows removal of any interposed intra-articular capsule, cartilage, or bone fragments, and the reduction can be confirmed under direct vision.^5^^,^ 15 A list of steps can be done to avoid the incision infection; care should be taken not to perform surgery on a foot with potential for skin compromise. Careful evaluation of the skin is performed at the first visit and again preoperatively. The time of the surgery is controlled strictly until the swelling dissipated and the sign of skin fold can be seen. Antibiotic is intravenously dripped thirty minutes before the operation, aseptic technique is applied, and a thorough debridement and syringe of the incision is needed. As for the dorsal plate fixation group, we have not encountered patient with deep infection which have to remove the implant, and we merely have encountered four patient with necrosis of incision edge and two superficial infection which were cured by oral antibiotic therapy and changing dressings.

Posttraumatic arthritis is a complication associated with Lisfranc joint injuries in approximately 30% of patients. However, it was recently reported incidence of osteoarthritis after ORIF of Lisfranc dislocations is 40% to 94%.^11^ These patients often require a conversion to an arthrodesis of the TMT joints.^19^ The incidence of arthritis is directly proportional to the area of damage on the articular surface. The iatrogenic destruction of tarsometatarsal joint surfaces cartilage maybe one of the reasons of post traumatic arthritis. Alberta's study showed in the specimens where transarticular screws were used, the area of visible articular surface damage caused by a single 3.5-mm screw varied from 2.0 to 4.8%.^16^ If we choose 4.0-mm screws for fixation, the amount of damaged articular surface may be larger. To this point, using a dorsal plate may avoid the iatrogenic destruction of articular surface. In our study the screw fixation group has six (21.4%, 6/28) patients who had spontaneous TMT joints fusion and fourteen (46.4%, 13/28) patients had degenerative joint disease according to X-ray, which is higher than plate fixation group which has five patients (15.6%, 5/32) who had spontaneous TMT joints fusion and eight (25.0%, 8/32) patients who had degenerative joint diseases.

Because of high incidence of osteoarthritis after ORIF of Lisfranc injuries, primary arthrodesis (PA) of severe Lisfranc injuries is advocated by some authors and their reports had better result than ORIF with screw fixation.^12^^,^ 13 However, it is still controversial and there is no report about comparisons between PA and dorsal plate fixation. We only take it as a secondary measure to treat posttraumatic arthritis in this study. In our study, the reoperation rate of PA in the plate group was 6.3%, while the reoperation rate of PA in the screw group was 10.7%. When we have such patients as severe Lisfranc injuries with comminuted fractures of metatarsals or cuneiforms, it may be an alternative to reduce long term sequelae. Is Myerson C type fracture-dislocation more fit to have primary arthrodesis than ORIF?

If we choose primary arthrodesis, fusion of which rays would have the best outcome according to varying injuries? These will be our objectives of study in next stage.

## CONCLUSIONS

Although there is still much controversy about how to treat Lisfranc injuries, the results of our study so far show that dorsal plate fixation for a dislocated Lisfranc injury do have better short and median term outcome and a lower reoperation rate than a standard screw ORIF. It may be a higher incidence of wound infections. In our experience, we recommend using dorsal plate in ORIF of a dislocated Lisfranc injury. Arthrodesis of TMT joints can be a supplementary measure for failed patients, a primary fusion maybe an alternative for some severe Lisfranc injuries.
